# Feedback Interventions in Motor Recovery of Lateropulsion after Stroke: A Literature Review and Case Series

**DOI:** 10.3390/brainsci14070682

**Published:** 2024-07-05

**Authors:** Maria Gomez-Risquet, Anja Hochsprung, Eleonora Magni, Carlos Luque-Moreno

**Affiliations:** 1 Facultad de Enfermería, Fisioterapia y Podología, Universidad de Sevilla, 41009 Seville, Spain; margomris@alum.us.es; 2 Unidad de Neurología, Hospital Universitario Virgen Macarena, 41009 Seville, Spain; anja.hochsprung.sspa@juntadeandalucia.es; 3 Instituto de Biomedicina de Sevilla (IBiS), Departamento de Fisioterapia, Universidad de Sevilla, 41009 Seville, Spain

**Keywords:** stroke rehabilitation, postural balance, gait disorders, neurologic, feedback, haptic technology, lateropulsion, pusher syndrome, contraversive pushing, pusher behavior

## Abstract

Lateropulsion is a post-stroke phenomenon marked by an active push of the body across the midline towards the more affected side and/or a resistance of the weight shift towards the less affected side. Within the mechanisms of treatment, feedback systems have been shown to be effective. The aim of the present study was to create a body of knowledge by performing a literature review on the use of feedback mechanisms in the treatment of lateropulsion and to report two cases of lateropulsion patients who had undergone feedback-based treatment. Methods: The review was performed across five different databases (Embase, Medline/PubMed, Scopus, Web of Science, and PEDro) up to February 2024, and haptic feedback intervention was incorporated into the case series (with lateropulsion and ambulation capacity as the main variables). Results: In total, 211 records were identified and 6 studies were included after the review of the literature. The most used feedback modality was visual feedback. In the case series, positive results were observed from the intervention, particularly in the recovery of lateropulsion and balance, as well as in the improvement of gait for one patient. Patients demonstrated good adherence to the intervention protocol without adverse effects. Conclusions: Visual feedback is the most commonly used feedback modality in lateropulsion patients but other mechanisms such as haptic feedback also are feasible and should be taken into account. Larger sample sizes, extended follow-up periods, and the isolation of feedback mechanisms must be established to clarify evidence.

## 1. Introduction

Stroke is the second cause of death worldwide and one of the main causes of disability [1]. Because of this, this pathology produces a high socio-sanitary cost, with repercussions on health systems as well as on patients’ and families’ lives. In particular, motor sequelae, specifically hemiparesis, stand out for their prevalence as the main cause of disability in these patients [1,2]. Within the cases where hemiparesis occurs, patients develop the so-called pusher syndrome (PS). Among the studies describing the prevalence of this condition, it has been established that 10% of cases with hemiparesis present it, while this percentage increases to 65% when the stroke is severe [3,4]. However, recent research highlights a higher prevalence of this pushing phenomenon regardless of its severity at greater percentages (55.1%) since it is now predominantly known as lateropulsion (and includes cases ranging from mild to severe impairment) [5]. The actual definition of this condition is “the phenomenon of actively pushing the body across the midline toward the more affected side, and/or actively resisting weight shift toward the less affected side”, regardless of underlying mechanisms. This aforementioned factor regarding the terminology of this entity must be considered because of the absence of a consensus in the literature [6]. Previously, terms such as “pusher behavior”, “pusher syndrome”, and “contraversive pushing” have been used to define this phenomenon [6,7]. 

This lack of agreement poses barriers to accurately comparing research findings, agreeing on measurement tools, establishing a consistent rehabilitation approach, understanding the prevalence of the condition, and applying research findings to clinical practice [7]. Although it might be presumed that the passage of time and the recent increase in research on this topic [8] may have favored the creation of a clear definition for this phenomenon, this has not been the case. In fact, in 2021, an expert panel based on the Delphi method began to address various aspects of lateropulsion, including terminological discussion. As a conclusion of the aforementioned report, it seems that the most appropriate term currently to refer to this clinical picture is lateropulsion [7]. Nevertheless, it remains a little-researched phenomenon, with studies with small sample sizes and not very rigorous designs [9]. Last year, Nolan et al. [6] established clinical recommendations for the management of patients with lateropulsion, as there was no evidence of the existence of any previous clinical practice guidelines. 

Among different interventions for post-stroke patients, feedback-based technology is an interesting and promising approach. It has been considered effective in fundamental processes of patient physiotherapeutic interventions such as gait re-education [10]. Feedback-based mechanisms provide information about the execution of an activity that the individual themself cannot consciously perceive [11]. This information is collected using specific instruments that process it and retransmit it through auditory, tactile, visual, electrical, or vibratory signals, among others [12,13]. In post-stroke patients with lateropulsion, visual feedback seems to be the most used type in physiotherapeutic interventions [14]. Despite this, various other types of feedback, such as auditory feedback, have also shown promising results in stroke patients [15,16,17]. 

Similarly, there is another type of feedback information, called haptic feedback. It is related to the transmission of information or experience through the sense of touch [18]. It is established as a way of providing sensory information to patients with sensorimotor disorders, involving neural structures similar to those activated during tasks in physical environments and facilitating motor learning through better sensorimotor integration [19,20,21]. Haptic feedback can be categorized into kinesthetic cues (which give spatial references to the user) and tactile cues (which include sensations like vibration, pressure, or texture), both of which can be transmitted through specialized devices [18]. This feedback is implemented in upper-limb recovery [22,23] and virtual reality environments [24]. In addition, these kinds of cues as feedback information have been previously used in balance training [25,26], body positioning [27], and gait re-education [18]. One of the ways in which haptic feedback can be provided to patients is through an electrical sensation on the skin; this can be categorized as a tactile stimulus, offering kinesthetic information to improve body control. 

In fields where feedback approaches are widely implemented (e.g., gait re-education), disorders of body perception or laterality are generally established as exclusion criteria [16,28,29]. Thus, patients with lateropulsion are excluded from most of the relevant studies on feedback-based therapies. In addition, recent reviews exploring therapeutic approaches for PS or lateropulsion have not established a specific research strategy incorporating feedback as a key component [14,30]. Consequently, our aim is to conduct a more focused examination of the literature and provide updated insights in the field up to the current year 2024, focusing solely on feedback mechanisms to ensure not to contaminate information provided. Our review encompasses an exploration of feedback mechanisms in these patients, supplemented by a case series involving two patients treated with haptic feedback therapy for lateropulsion. Thus, the primary objective of this study is to contribute to the existing knowledge base on feedback techniques in the clinical management of lateropulsion. Additionally, we highlight the feasibility of haptic feedback as a potential treatment modality through the presentation of a case series. 

## 2. Materials and Methods

### 2.1. Research Protocol

A literature review has been performed, searching all articles referring to feedback-based techniques used in the treatment of pushing or lateropulsion patients. Moreover, a case series concerning two patients with lateropulsion and a haptic feedback treatment was performed. The PRISMA 2020 statement was followed in the structure of the review in [31] and the CARE Checklist was used as a framework for the case series, as well as the National Institutes of Health (NIH) quality assessment tool for case-series research [32,33]. 

### 2.2. Search Strategy, Eligibility Criteria, and Data Extraction

The review was performed by searching for potentially eligible trials up to February 2024. This was conducted by two independent reviewers (M.G.-R. and E.M.) and an extra reviewer (C.L.-M.) was consulted for consensus as necessary. The search of the studies was performed in five different databases: Medline/PubMed, Scopus, Web of Science, Embase, and PEDro (search strategies available in Table 1). The PICOS criteria [34] included the following: P (Population): post-stroke patients with lateropulsion, PS, pusher behavior, or contraversive pushing; I (Intervention): feedback; C (Comparator): other techniques or no comparison; O (Outcome): measured through specific and validated tests or scales related to the International Classification of Functioning (ICF); S (type of study): clinical trials (controlled and non-controlled, randomized and non-randomized), pilot studies, case reports, and case series. The research strategy included all available records in English and Spanish and the results were filtered to these languages. Exclusion criteria included interventions where feedback was part of robotic mechanisms, as these could mask the real impact of the feedback since these mechanisms are typically very powerful intervention methods. 

For the selection of articles, we implemented a rigorous three-step procedure. The first step involved thorough database searches and reviews of titles and abstracts. In the second step, articles were excluded based on their title or abstract, with further analysis conducted against predefined inclusion criteria. The third and final step entailed a comprehensive examination of the full text of each eligible article.

### 2.3. Case Series

#### 2.3.1. Patients Included and International Classification of Functioning Diagnosis

A series of two cases was carried out in a pre-experimental procedure as a pilot phase of the research protocol. This study was conducted in accordance with the Declaration of Helsinki and approved by the Research Ethics Committee (CEI) of the Virgen Macarena and Virgen del Rocío University Hospitals (code 2022-03; Seville, Spain). 

A 52-year-old man (case 1) and a 69-year-old woman (case 2) were included in our case study. Both presented lateropulsion after stroke and were admitted to Virgen Macarena’s Hospital in Seville. Baseline characteristics and clinical features of the participants are showed in Table 2. 

ICF diagnosis [35] can be used to describe and classify health and health-related conditions and has been highlighted as a necessary tool for providing a holistic and patient-centered approach [36]. Case 1 exhibited marked muscular weakness in the trunk (b7305.2), as well as hemiparesis on the right side of the body. In the right upper limb, there was complete muscular and sensory impairment (b299.4, b7301.4), while in the right lower limb, there was some activity in the hip flexor muscles (b299.4, b7301.3). Lateropulsion was significantly pronounced (b799.3), and there was a lack of coordination in complex voluntary movements (b7602.4). He was unable to perform transfers from a supine state to a sitting state or from sitting to standing (d410.4), although he was able to maintain a sitting posture, at least for short periods of time (d4153.2). He could not maintain standing balance (d4154.4) or walk (d4509.4). There was no pain (b280.0). In terms of functional independence, he was only independent in eating (d550.0), while for other activities (washing, dressing, toileting), he required assistance. He had fecal and urinary continence (b5253.0, b6202.0). There was family support (e310+4). 

Case 2 presented significant muscular weakness in the trunk (b7305.3), and hemiparesis on the left side of the body. Complete muscular and sensory impairments were observed in the left upper limb (b299.4, b7301.4), and partial impairments in the lower limb of the same side, showing muscle activity in the hip extensors (b299.4, b7301.3). Lateropulsion was very pronounced (b799.4), and there was a lack of coordination in complex voluntary movements (b7602.4). She also was unable to perform transfers from a supine state to a sitting state or from sitting to standing (d410.4), although she could maintain a sitting posture, at least for short periods of time (d4153.2). She could not maintain standing balance (d4154.4) or walk (d4509.4). She experienced pain (b280.3). In terms of functional independence, she was only independent in eating (d550.0), while for other activities (washing, dressing, toileting), she required assistance. She had fecal and urinary continence (b5253.0, b6202.0). Finally, she had family support (e310+4) but faced housing space limitations (e1551.4).

#### 2.3.2. Treatment Approach

**Figure 1:**
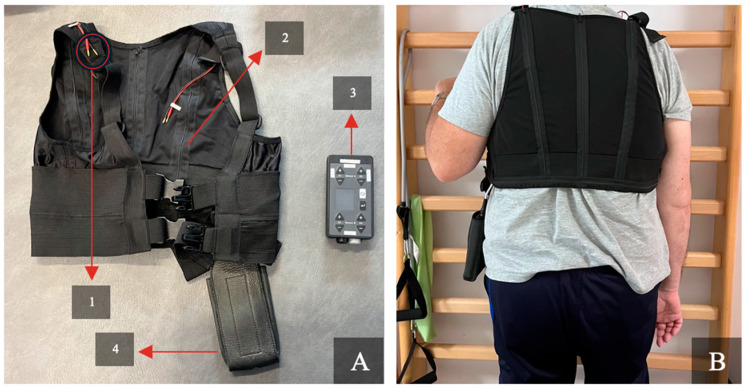
(**A**) Walking Aid ReMoD V5.0 Type 1 (vest and device). 1: Connection cables that transmit the signals, and where the electrodes are plugged in (the electrodes are then placed on the patient’s skin to provide stimulation). 2: Sensors integrated in the vest. 3: ReMoD V5.0 Type 1 control unit device, which provides the electrical stimulus (it features multiple buttons for setting and controlling the signal intensity). 4: Pouch for storing the device while it is in use. (**B**) Case 1 standing autonomously with the ReMoD vest in a session.

In the physiotherapeutic approaches to these patients, the Walking Aid ReMoD V5.0 Type 1 (ReMoD UG, Berlin, Germany) was used (Figure 1). This aid comprises motion sensors, signal transmitters, and a control unit integrated into a vest tailored to the patient’s body. The system delivers haptic electrical stimuli when the trunk bends beyond pre-established degrees, providing real-time feedback to the patient. The electrical stimulus is delivered via two electrodes placed at the infraclavicular and supraclavicular points on both shoulders of the patient.

The intervention included six physiotherapy sessions within two weeks and extra time to use the vest autonomously (almost three hours a day). The sessions took place between the hospital and the patients’ residences upon discharge. The physiotherapeutic intervention consisted of mobility, postural, and motor control exercises. Exercises to improve balance, transfers, and lower-limb strength and gait training were also performed. An approach based on the performance of cognitive tasks in some exercises was used to improve the acquisition of motor patterns. Both patients performed the exercise protocol with the ReMoD device so that they received feedback about their trunk tilt during the session. This allowed better control of the trunk and continuous provision of information about verticality. 

#### 2.3.3. Outcome Variables and Evaluation

In this study, lateropulsion and ambulation capacity have been established as the main outcome variable. On the other hand, the rest of the dependent outcome variables have been considered secondary. The scales and tools used to measure lateropulsion were the Scale of Contraversive Pushing (SCP) and the Burke Lateropulsion Scale (BLS). The SCP has 3 components: 1, symmetry of spontaneous body posture; 2, use of limbs (legs and arms); and 3, resistance to passive correction of tilted posture. The total SCP score ranges from 0 to 6, with a higher score indicating a more severe presentation of PS [37]. Originally, patients who met at least one point in each component of the SCP were classified as pushers, but other authors have recently sought to broaden these scoring criteria to include patients with milder lateropulsion (with a total SCP score > 0.5 and at least one of the three components of the SCP with a score < 1) [38]. On the other hand, the BLS has five items of pushing behavior that are assessed in different positions (supine, sitting, standing, walking, and during transfers). It achieves a higher score as the resistance increases across the various items (total score from 0 to 17) and the diagnosis of pushing behavior is considered when two or more points are obtained [39]. Both scales have been studied and exhibit good inter- and intra-observer reliability [37,39].

For the measurement of balance, the following scales were used: Firstly, the Postural Assessment Scale for Stroke (PASS) assessed postural control [40]. This scale consists of 12 items that score according to lower or higher levels of functionality, with a total score of 36 points [41]. The Trunk Impairment Scale (TIS) was also used to assess balance and coordination, where the maximum score achievable is 23 (with higher scores indicating greater trunk mobility) [42,43]. Finally, the Performed Oriented Movement Assessment (POMA) assessed stability tasks related to daily activities and gait performance. This has a maximum score of 28 points and determines a person’s fall risk (18 points or lower) [44]. 

To assess pain, the short form of the McGill Pain Questionnaire (SF-MPQ) was used. In this questionnaire, pain descriptors are categorized into three dimensions (sensory, affective, and evaluative) and the overall score is the result of counting the words selected by the patient and their severity [45]. The total score is 45 points, awarded when severe pain is presented in all descriptors [46]. Related to walking capacity, walking 50 m was measured [47,48] (with or without assistance). The Functional Ambulation Category (FAC) was also used to quantify each patient’s ambulation capacity within a 5-point scale (0 = “absolute inability to walk” and 5 = “walking normally for unlimited distances”) [43]. 

On one hand, the Modified Rankin Scale (MRS) was used to measure the degree of functional independence [49,50]. It has 7 scores (0 corresponds to no deficit and 6 corresponds to death) [50]. The Barthel Index also measured independence: this index consists of 10 items exploring activities of daily living, with a maximum score of 100 meaning maximum independence [51]. On the other hand, the Quality of Life Scale for Stroke (Spanish scale ECVI-38) and the Hospital Anxiety and Depression Scale (HADS) were used in the quality of life and depression assessments. ECVI-38 measures quality of life. This scale comprises 38 items grouped into 8 domains, with a 5-point scale employed for each item (5 points as an extreme condition). The total score is calculated based on the average of the domains, with a maximum of 100% [52]. The HADS assesses depression and anxiety within 14 items. Possible scores for each item range from 0 to 3, and greater symptom severity is evidenced when items exhibit higher values [53]. 

A total of three assessments were performed on the two participants: a baseline assessment before the start of the intervention (pre-test assessment), a final assessment after the intervention ended (post-test assessment), and a follow-up assessment one month after the intervention. 

## 3. Results

### 3.1. Selection of Studies for the Review

**Figure 2:**
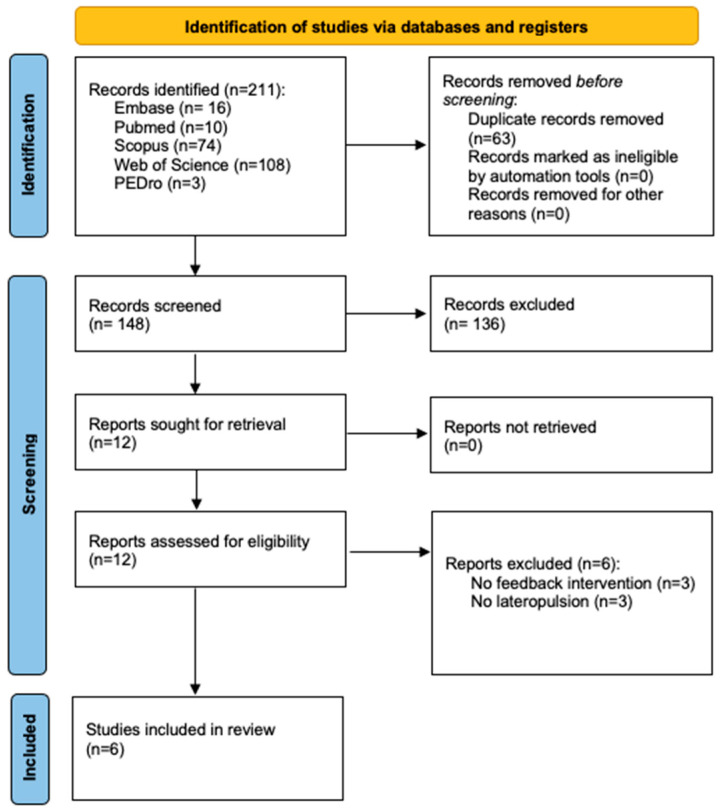
PRISMA 2020 flow diagram for new systematic reviews, which included searches of databases and registers only [31].

Of a total of 211 articles identified after the screening procedures, only 6 met the inclusion criteria. In Figure 2, the process flow diagram is shown. In Table 3, a summary of the main characteristics of the studies included and their interventions is presented.

### 3.2. Results from the Review

#### 3.2.1. Lateropulsion

The SCP and BLS were included in five studies [55,56,57,58,59] and one study [57], respectively. Improvement in the SCP was either related to feedback conditions [56,58,59], had no relation to feedback conditions [55], or did not show relevant results compared to other interventions [57]. In the case of the BLS, one study reported improvement [57].

#### 3.2.2. Balance

Two studies measured this variable with the Berg Balance Scale (BBS) [54,56]. Visual feedback explained the improvement in BBS scores in one study [54]. In this study, carried out by Yang et al. (2015) [56], both feedback conditions (computer-generated and mirror feedback) showed positive results in this scale, but there was significant improvement in the experimental group. On the other hand, the PASS was also used in one other study to assess balance [55], reporting an increase in outcome scores after feedback training. Additionally, the Modified Clinical Test of Balance (mCTSIB) carried out by Nakamura et al. [54] reported better results after phase B of their intervention design. 

#### 3.2.3. Motor Function

Two studies measured this variable [56,59] with the Fugl-Meyer Assessment Scale (FMA), showing an improvement in both feedback and no-feedback conditions. Paci et al. [59] also used Lindmark’s assessment to evaluate this outcome and found positive results after intervention.

#### 3.2.4. Functional Independence

The Barthel Index gave information related to this variable in two articles [55,59]. Both articles showed an improvement in this outcome.

#### 3.2.5. Other Outcomes

The ataxia of one patient was assessed in one study using the Scale for the Assessment and Rating of Ataxia (SARA) [54], presenting positive results. Other variables, such as the excitability of the vestibulospinal tract (VST) or the perception of visual vertical and the imbalance in the vestibular system, were analyzed using GVS and transcutaneous stimulation and the Subjective Visual Vertical (SVV) approach, respectively [54]. Also, the Modified Ashworth Scale (MAS) was used in one study [59], with no relevant results. 

### 3.3. Results from the Case Series

The results of the pre-test, post-test, and follow-up assessments are shown in Table 4. During the implementation of the case series, there were no adverse effects in the participants, and they showed very good adherence to treatment.

In the pre-test evaluation, the patients had similarities in their SCP, BLS, TIS, and POMA scores. It is notable that the participants differed greatly in the results of the PASS (where patient 1 had a score of almost twice as much as patient 2’s score). In addition, neither of them had the capacity to walk 50 m before the intervention and had equal scores in the FAC. The patients also differed in their SF-MPQ scores (only participant 2 showed pain during the pre-test). The MRS and BI results were also similar, but it is important to note that both patients exhibited differences in their initial scores for the ECVI-38 and HADS. 

After the intervention, improvements were observed in the SCP and BLS scores for case 1, while case 2 showed only a decrease of one point in her SCP score and did not exhibit improvement on the BLS. There were enhanced scores on the PASS for both participants. Additionally, case 1 demonstrated improvement in his TIS and POMA scores. The TIS score for case 2 also improved, but on the POMA scale, this patient only scored one point more. Regarding ambulation ability, case 1 achieved the ability to walk 50 m post intervention, whereas case 2 did not. Nevertheless, the results on the FAC scale for both patients were modest. The SF-MPQ scores for patient 2 showed a reduction of almost half compared to the pre-test assessment. Neither of the patients showed improvement on the MRS following the intervention. In contrast, both cases demonstrated an increase in their BI scores. Finally, while case 1 showed better results on the HADS, case 2 exhibited an increase in her score, indicating higher levels of anxiety and depression.

In the follow-up assessments, case 1 had fully recovered from his lateropulsion and case 2 showed an improvement in her SCP score (from 5 points to 2.25). Both patients showed improvement on the PASS and case 1 also experienced good progress in his TIS and POMA scores. Other notable findings included complete pain recovery in case 2, as evidenced by her SF-MPQ scores.

These results are based on the increases shown in the scores of the scales, but it is important to note that these scores should always be contextualized according to the maximum score of each assessment instrument. Also, it will be of great interest to pay attention to the internal constructs of each tool. In addition, there are not enough data to support associations in a statistically significant way.

## 4. Discussion

### 4.1. Discussion Related to the Review

After conducting this specific research in the field of feedback interventions for lateropulsion patients, visual feedback emerges as the predominant approach. These results are consistent with previous studies [14], although a more detailed discussion of specific aspects derived from these results is provided below. While the reviewed studies support this mechanism as an intervention, some studies did not prioritize it at the center of their treatment strategy and used visual feedback as a complementary tool. However, others focused more on it, including the use of computer-generated interactive feedback [56], the use of the Subjective Postural Vertical (SPV) approach [55], or Broetz et al.’s intervention (in general terms, their approach focuses on sensory reorientation based on exploring visual surroundings and the correction of postural perception through specific exercises aimed at promoting body awareness and improving balance control) [58]. Nonetheless, concerns exist regarding the sole reliance on visual feedback due to potential patient impairments [38,60], and multimodal approaches are recommended to address these issues [6]. However, the results of these mixed intervention approaches do not provide clear evidence of the efficacy of the feedback mechanism itself, with the establishment of methodology being crucial in these cases.

Only one study reported explicitly another type of feedback, the auditory one, employing vocal feedback from a therapist [59]. Despite this, auditory–vocal feedback is a practically implicit approach in physiotherapeutic interventions. In contrast, recent advancements have proposed various auditory feedback mechanisms for post-stroke patients. These mechanisms are normally based on the provision of auditory information related to weight transfer, used, for example, in gait retraining [61,62,63,64,65]. Future research should investigate which auditory mechanisms related to weight transfer could aid in lateropulsion recovery. Additionally, it will be necessary to increase the utilization of technology across all interventions with lateropulsion patients, thereby aligning the progress in this field with current advancements in physiotherapy [66].

The interventions in the reviewed studies included balance and weight-transfer training [54,56,57,58,59], gait training [54,57,59], SPV training [55], the Bobath concept and specific lateropulsion activities [59], galvanic vestibular stimulation (GVS) [57], and other fundamental and specific principles based on the Broetz and Karnath approach [58]. GVS emerges as a distinctive intervention with differing opinions in the literature [57,67], but it is not mentioned in current clinical practice evidence [6]. Finally, even though more than half of articles [54,56,57,59] included gait as a part of their interventions, no results for this outcome variable were reported as there was no measure established. 

Variability was observed in the methodology outcome variables, outcome measures, and interventions among the selected studies meeting the inclusion criteria of this review. Nakamura’s study [54] had the highest number of distinct clinical scales, potentially due to their classification of patients as non-pushers. This diagnostic was based solely on the results of the BLS (which, as previously mentioned, only focuses on resistance and not on the other potential characteristics of a patient with lateropulsion). However, they incorporated the patient’s syndrome (body lateropulsion) into the lateropulsion category as a collective entity. The terminological issue has been emphasized several times in this article, and this recent study is a great example of the extent to which classification controversy still exists. In fact, some authors distinguish between “lateropulsion” (a tilt) and “pushing behavior” (active pushing that results in a tilt) [7]. The disagreement concerning the active versus passive characteristics of this condition likely hinders the act of reaching a consensus [7]. This study could be a reflection of that.

#### 4.1.1. Lateropulsion

Five studies included the SCP and only one included the BLS [57]. The literature is scarce regarding values of minimal clinically important difference (MCID) for these scales [68]. MCID is a threshold value that represents a change perceived and detected by the patient after an intervention, and it is distinct from statistically significant difference [69]. It is important to correlate statistically significant results with their clinical impact to avoid misinterpretations of study findings, which may lead to unnecessary patient therapy exposures [70].

Two out of five studies exploring lateropulsion demonstrated favorable results for lateropulsion following visual feedback treatment. Yang et al. [56] showed better results with an interactive visual feedback approach compared with using conventional visual feedback. Notably, this study was the only randomized clinical trial included in this review that provided pre-/post-training results, with effects being observed after a longer intervention rather than immediate effects. Consequently, the findings of this study warrant high consideration. The enhancement in the experimental group could be attributed to the engagement with the program and the informative feedback regarding various movement planes. Broetz et al. [58] reported modest recovery from lateropulsion, with patients only achieving autonomous sitting post intervention. This might be attributed to the short duration of sessions and, in particular, variations in the total intervention duration among patients. One important difference between these two studies [56,58] is the time post stroke. One conducted its intervention in a very acute phase (average of 4 days) [58], while the other worked with patients with subacute and chronic stroke [56]. Thus, the modest improvement reported by Broetz et al. [58] could be relative to the early phase that the patients were in at the time of inclusion.

In contrast, Lee et al. [55] generally found better results in lateropulsion after an intervention without visual feedback compared to those after one with visual feedback, potentially due to there being a quieter environment during the visual feedback sessions. It is important to note that environmental factors like noise and lighting can affect the effectiveness of auditory or visual cues. However, caution is needed in interpreting these results due to the intervention alternation and potential carry-over effects of the study. Krewer et al. [57] did not report significant improvements after visual feedback intervention compared to driven gait orthosis. Moreover, this study did not show statistically significant differences between these interventions according to the SCP but did according to the BLS. Thus, the interventions’ effects were mainly reported regarding resistance rather than encompassing all signs of lateropulsion included in the SCP. Finally, Paci et al. [59] reported immediate but not lasting improvements in SCP results after visual and auditory feedback therapy. However, due to the lack of separation between different procedures in this intervention, it is challenging to attribute specific results to each one.

#### 4.1.2. Balance

The BBS was used in two studies [54,56]. In both studies, platforms were used: either as a measurement tool [54] or within the intervention [56] (force platform and Nintendo Wii balance board, respectively). Also, information about the center of pressure (COP) was used in both designs. In the study of Yang et al. [56], a statistically significant improvement in balance for the experimental group was observed. The study by Nakamura et al. [54], being a case report, did not report statistical information for this result but did report an improvement. In another recent study [71], the MCID established for the Berg Balance Scale in subacute stroke for assisted walking patients was of 5 points. This MCID for this scale was reached in both aforementioned studies. Nakamura et al. [54] also used another test to assess balance: the mCTSIB, wherein COP positions and velocities were measured. An increased velocity of COP was reported to be associated with abnormal postural control, and when given postural inputs, the patient reduced this velocity. The cause of this, as mentioned by the authors, could be the reduction in voluntary movements. Nevertheless, this case report’s design requires caution when interpreting the results. Also, the setting of different treatment phases in this study would have some carry-over limitations, like in the study reported by Lee et al. [55].

Lee et al. [55] used the PASS to measure balance after intervention (SPV approach). Their study reported better results after visual feedback training. However, according to the established MCID scores for this scale (3 points) [72] for stroke patients, only one patient would have achieved this difference (patient 1), and in both clinical settings (with and without visual feedback). Although in this study the mean improvement across the three patients was greater for the intervention without visual feedback, it is important to consider these clinically significant differences for patient 1. Nonetheless, caution is advised when interpreting the results of this study due to the aspects of its intervention methodology exposed before.

After these interpretations, it could be considered that in terms of weight shifting and awareness of weight information interventions, the results regarding balance are good when using visual feedback. In contrast, with other approaches, such as the SPV approach, studies report better results in balance for patients deprived of this information.

#### 4.1.3. Motor Function

This outcome variable was measured in two studies with the FMA. On one hand, Yang et al. [56] reported significant improvement for this scale in both groups (experimental and control), but without significant difference between them. This shared improvement could be related to their use of weight-shifting training with a balance board. In upper extremities, the authors did not report good results, as the aforementioned treatment primarily targets posture control, neglecting upper-limb involvement. On the other hand, Paci et al. [59] showed functional recovery with an improvement in the FMA of 14 points. Latest reviews have established that an improvement of between 4 and 12.4 points in the FMA would be considered an MCID for stroke patients [73]. In this case, both studies achieved this threshold. Paci et al. [59] also included a motor assessment according to Lindmark’s method and achieved an improvement of two points after their intervention. 

#### 4.1.4. Functional Independence

Only two articles [55,59] provided information about this variable using the BI. Due to their methodological designs (case series and case report, respectively), they did not report their results with a statistical analysis. According to results and the MCID threshold established for the BI in stroke patients (from 4 to 5 points) [74], both studies achieved an MCID. In Lee et al.’s study [55], patients surpassed this MCID after both interventions (with and without visual feedback), even though higher percentages of improvement were reported for the training without visual feedback. Furthermore, patient 1 in this study exhibited the greatest recovery, achieving an average increase of 11 points between the interventions. Existing research has consistently shown greater recovery rates among male patients [75], as well as a correlation between functional independence and younger age [76]. Notably, this patient was the youngest among the three participants enrolled in the study protocol. 

Although both studies showed positive results, there is variance in the improvement reported in BI scores between the study by Paci et al. [59] and the study by Lee et al. [55]. The score of the patient from the first study improved more than twice (30 points) as much as that of the patient in the second study. This might be attributed to differences in the intervention duration. Specifically, the intervention duration was nearly twice as long in Paci et al.’s study [59] compared to Lee et al.’s study [55]. Thus, a possible dose–response effect could be hypothesized, and it might be possible that a minimum number of sessions could be necessary for improvement in this variable in future studies.

#### 4.1.5. Other Outcomes

The SARA was used in Nakamura’s study [54] due to the patient presenting ataxia, and after intervention, there was a great improvement for this outcome. Also in this study, the excitability of the VST was assessed through GVS and transcutaneous stimulation of the tibial nerve to evoke the ipsilateral soleus H-reflex. Although there was improvement in postural control, the VST excitability did not change on the affected side. While Krewer et al. [57] used GVS as a therapy, Nakamura et al. [54] used it to study the underlying cause of body lateropulsion in their study. They also used the SVV test to assess the patient’s perception of visual vertical and the imbalance in the vestibular system but did not report results for it. Other outcome measures such as the MAS were used in the study of Paci et al. [59] in the knees and wrists of the patient. However, although the patient was assessed, there was no increase in tone observed either before or after the intervention.

### 4.2. Case Series Discussion

In this case series, we explore an alternative feedback intervention for stroke patients. Some previous studies have used haptic feedback and vibrotactile cues to enhance balance and gait recovery in healthy populations and stroke patients [18,27]. However, the proposed mechanism is novel due to the type of stimulus, provided to the patient through electric sensations on the skin. Furthermore, its easy adaptation to the patient’s body allows for its appropriate use both in clinical settings and outside of them. Our intervention also relies on a therapist support strategy. As some previous studies have pointed out, it is challenging for a therapist to remain alert to all necessary corrections in their approach to lateropulsion patients in order to properly develop motor patterns [77]. The haptic feedback implemented in this case series serves as an aid to complement verbal feedback based on commands and it assists the therapist in correcting other motor aspects during the intervention. In addition, the implementation of this intervention in the acute stage for both patients aligns with findings in the existing literature, emphasizing the need for early intervention [78]. Patients with post-stroke lateropulsion typically take approximately 3.6 weeks longer on average to achieve recovery, and even longer to achieve functional independence [9]. Therefore, the therapeutic approach developed in this case series and its use in the early stage addresses this aspect as it can be used in seated positions (in contrast to other device designs that require patients to stand [15]).

Related to the implementation of specific scales, we have used both the SCP and the BLS, as having as much information as possible is beneficial for clinical practice. Both patients showed improvements in lateropulsion throughout the process (with case 1 reaching full recovery). However, further studies on the psychometric properties of these scales are needed to detect the MCID for them and to interpret this type of results more clearly. Nevertheless, all data resulting from our intervention should not be generalized due to the established study design. 

The associated disruption in activities of daily living that causes the impairment of trunk and gait [43,76] made these last two outcome variables the focal points of this case series. That is why we aimed to approach trunk impairment and balance more comprehensively, using scales such as the PASS or TIS, and to assess walking capacity using various methods. The value assigned as the MCID for the PASS according to recent studies is 3 points [72], and for the TIS in patients with acute stroke it has also been established at 3 points [79]. Based on these data, it can be confirmed that both participants exceeded these two thresholds after the intervention. In addition, other studies have linked the results of these two scales (PASS and TIS), highlighting a significant association [43]. This aspect aligns with the results obtained for case 1, although there are not enough data to statistically support these associations. It is also important to highlight the relationship that certain studies have emphasized between the TIS and gait independence, establishing the former as a predictor of the latter [80]. Also, a connection between age and scores in the TIS has been established, linked with a better prognosis in gait recovery [80]. It can be observed that participant 1, with higher scores on the TIS and a lower age, showed a faster and more functional improvement in gait (he achieved the ability to walk 50 m with an orthotic assistance). However, it is important to note that these associations have been made based on studies with stroke patients but without lateropulsion, so they must be interpreted with caution. Also, it must be considered that the better recovery of participant 1 could be related to the type of stroke. Despite causing more mortality [81], evidence indicates that hemorrhagic strokes show greater functional improvement in recovery processes compared to ischemic ones [82]. Finally, it is crucial to emphasize other positive aspects of the intervention, like the decrease in pain experienced by participant 2.

Throughout the study, our aim was to adopt a patient-centered approach, considering both the patient and their biopsychosocial environment, in accordance with the ICF [35]. The importance of this holistic approach has been emphasized in both the methodology and results of the present study. Additionally, within the constructs of participation, activity, and environment, scales such as the MRS, BI, ECVI-38, or HADS have been used with this intention. We believe that it is essential that they are considered within the process of recovery of the patient. For example, studies have highlighted cumulative incidences reaching up to 52% for post-stroke depression in the first five years following the event, as well as a pooled prevalence of 29% that persists even after ten years post stroke [83]. Over the past two decades, there has been a growing focus on holistic healthcare with the patient at the center, facilitated by key frameworks such as the ICF and the capability approach [36]. In the assessments taken one month after our intervention, the scores on the HADS remained high for both participants. Therefore, this aspect is an important point to consider, as reducing these scores and working within the aforementioned holistic framework were objectives of our intervention. The age factor, previously identified in the literature as a handicap in recovery, particularly in the early phases [76], may have influenced the recovery process for case 2. Additionally, concerning gender, certain studies have highlighted that women seem to have a lower likelihood of attaining complete functional independence and are more prone to disability following a stroke compared to men [75]. The results for the MRS, BI, or HADS in case 2 align with what has been highlighted from the literature.

### 4.3. Limitations of this Review and Case Series Study

The reviewed studies and the presented case series have several limitations. In the reviewed articles, small sample sizes, a lack of follow-up results (except Nakamura et al.’s study [54]), multiple intervention approaches [54,55,59], uncontrolled designs [54,55,57,58,59], and different intervention doses are the main limitations. Some of these limitations are also observed in the case series. The aforementioned mix of different types of approaches used in some studies should be highlighted: there is a limitation when feedback is merely a tool within the developed intervention and not the main intervention. This goes against the comprehension of specific results and their relationship with the feedback methodology. Specifically, in the present case series, the discharge of the patients from the hospital was a limitation, as sometimes continuing sessions at home may not be as efficient as continuing treatment in the hospital due to inadequate resources and infrastructure. This limitation has also been studied in recent research [84] and was an aspect that greatly complicated the treatment of participant 2, as she had serious space constraints in her home (ICF e1551.4). This could have influenced the results we obtained. Another aspect to consider is the difference in post-stroke time between both patients included in this case series. However, both were in the early stages of the condition and had very similar baseline characteristics. The acute phase of both patients may have been a limiting factor due to the possibility of spontaneous recovery. However, the proposed approach has been grounded in evidence that highlights the importance of early intervention in these patients [9,78].

Additionally, there have been some limitations related to the search strategy process. Firstly, the terms “pusher” and “feedback” can also be related to other fields, such as engineering or computer science. This aspect caused confusion and slowed down the reading and synthesis of results in the conducted search. Secondly, the present review focused on searching for interventions based on feedback. Some robotic or mechanized proceedings, such as those used in gait recovery, sometimes include this type of feedback intrinsically [85]. Nevertheless, as specified in the exclusion criteria, in these cases the true reason for improvement could not be assessed (as robotics could have confounded the true effect of the feedback, as they are generally very potent intervention mechanisms). In addition, information may have been lost about studies where visual feedback had been established as a control intervention or had been explained without any detail or as an optional strategy.

## 5. Conclusions

Although it is a little-researched condition, research on lateropulsion has recently experienced an increase in publications. Visual feedback has been, to date, the most used feedback mechanism in approaches to this condition. However, new approaches such as haptic feedback, as presented in this case series, also appear to be interesting and have yielded positive results. Following our intervention, no patient experienced adverse effects and good adherence was demonstrated. Additionally, positive results were observed from the intervention, particularly in the recovery of lateropulsion and balance, as well as in the improvement of gait for one patient. Nevertheless, all of these findings need to be interpreted within the context of the implemented methodological design of a two-patient case series, and they should be thoroughly investigated in future studies. In general, current technology in feedback systems (auditory, haptic, visual, etc.) still needs to advance in treating this condition. These technologies would not only provide patients with information on movement execution or spatial positioning but also would also support physiotherapists in better controlling outcomes during treatment sessions. Generally, interventions are focused on balance, weight shifting, gait retraining, and other specific procedures. Research with larger sample sizes and longer follow-up periods, as well as research that further isolates different feedback approaches for greater clarity in results, is a need that arises from the discussed limitations. This need can be turned into very interesting future research improvements.

## Figures and Tables

**Table 1:** Search strategies for different databases.

Databases	Search Strategy
Embase	feedback system AND (lateropulsion OR “pusher syndrome” OR “pusher behavior” OR “contraversive pushing”)
Medline/PubMed Web of Science Scopus	feedback AND (lateropulsion OR pusher OR “pusher syndrome” OR “pusher behavior” OR “contraversive pushing”)
PEDro	“Pusher behavior”, “pusher syndrome”, “contraversive pushing”, and “lateropulsion” combined with “feedback” as keywords (simple search)

**Table 2:** Baseline characteristics and clinical features of the case study participants.

	Case 1	Case 2
Sex	Male	Female
Age	53	69
Type of stroke	Hemorrhagic	Ischemic
Hemisphere affected	Left	Right
Time post stroke (days)	33	6

**Table 3:** Summary of the main characteristics of the studies included.

Authors (Year)	Study Type (n)	Age (Years)/TPS	Intervention	Dose	Outcome Measurements	Results
Nakamura et al. (2023) [54]	CR 1	67/ 48 days	3 interventions phases: A: Balance and gait training with VF. B: Exercises focused on SI (loads, closed eyes, etc.) and balance and gait training. FU: balance and gait training without VF or SI	9 sessions (1 h)/12 days. Total of 9 sessions	SARA, BBS, mCTSIB (force platform), H-reflex from soleus, VST excitability, SVV	SARA and BBS improvement occurred mainly after phase A. Generally better performance of mCTSIB after phase B and FU. VST excitability did not change on the affected side. SVV did not change significantly throughout the study.
Lee et al. (2017) [55]	CS 3	61.67/ 2.3 months avg.	Routine PT, SPV VF+, and SPV VF−. Alternating treatments with multiple baseline measures	3 sessions (1 h)/week. Total of 18 sessions	SCP, PASS, BI SCP-b = 5	Relative to baseline, all interventions showed improvement. BI, SCP, and PASS scores improved after SPV VF− training compared to after SPV VF+ training in a longer intervention stage.
Yang et al. (2015) [56]	RCT 12 (EG = 7; CG = 5)	60 ± 15.1/5.9 ± 3.65 months	Computer-generated VF (via Nintendo Wii balance board) or mirror VF	3 sessions (20 min VF + 20 min PT)/week for 3 weeks. Total of 9 sessions	SCP, BBS, FMA, SCP-b = 4.65 ± 1.05	Both interventions were associated with decreased lateropulsion and improvement of balance (significant difference between groups in favor of experimental: *p* < 0.01 and *p* < 0.05, respectively).
Krewer et al. (2013) [57]	RCT (cross-over) 25 (15 pusher and 10 non-pusher)	65.5 ± 9.5/ 7.7 ± 6.9 months	GVS, DGO, and PT-VF	1 single session of each type. Total of 3 sessions	SCP, BLS, SCP-b = not found	No statistically significant difference between interventions according to SCP. BLS results showed significant improvement after DGO compared to after PT-VF (*p* < 0.05). Other comparisons did not show significant difference.
Broetz et al. (2004) [58]	CS 8	63 avg./ 4 days avg.	VF (exploring surrounding vertical features)	6 sessions (30 min)/week within 26 days approx. Total of 22 sessions approx.	SCP, SCP-b = not found	Lateropulsion improved significantly after 3 weeks (*p* < 0.05). At day 24, six patients sufficiently recovered, achieving the sitting position unsupported (*p* < 0.05).
Paci et al. (2004) [59]	CR 1	71/ 27 days	Bobath concept, specific pushing activities (somatosensory inputs), and AF and VF (line in mirror)	6 sessions (2 h PT twice a day from Monday to Friday and 1 h on Saturdays)/week. Total of 27 sessions	SCP, FMA, MA, BI, MAS, SCP-b = 4.75	Immediate effects after feedback but not after somatosensory approach. No maintenance of these effects to the end of treatment. Lateropulsion was reduced only partially.

Abbreviations: AF: auditory feedback; avg.: average; BBS: Berg Balance Scale; BI: Barthel Index; BLS: Burke Lateropulsion Scale; CG: control group; CR: case report; CS: case series; DGO: driven gait orthosis (Lokomat); EG: experimental group; FMA: Fugl-Meyer Assessment Scale; FU: follow-up; GVS: galvanic vestibular stimulation; MA: motor assessment (Lindmark’s); MAS: Modified Ashworth Scale; mCTSIB: Modified Clinical Test of Balance; PASS: Postural Assessment Scale for Stroke Patients; PT: physiotherapy; RCT: randomized controlled trial; SARA: Scale for the Assessment and Rating of Ataxia; SCP: Scale of Contraversive Pushing; SCP-b: SCP score at baseline; SI: somatosensory information; SPV: Subjective Postural Vertical; SVV: Subjective Visual Vertical; TPS: time post stroke; VF: visual feedback; VF+: with visual feedback; VF−: without visual feedback; VST: vestibulospinal tract.

**Table 4:** Synthesis of results from the case series.

Results			Case 1			Case 2		
ICF	Variable	Scale/Test	Pre-Test	Post-Test	Follow-Up	Pre-Test	Post-Test	Follow-Up
Body function (b)	Lateropulsion	SCP	5.75	1.75	0	5	4	2.25
		BLS	11	3	0	10	10	5
	Balance	PASS	12	20	26	7	12	17
		TIS	2	16	17	2	8	8
		POMA	0	8	12	0	1	1
	Gait	50 M	No	Yes	Yes	No	No	No
		FAC	0	1	1	0	0	1
	Pain	SF-MPQ	0	0	0	14	6	0
Activity and Participation (d)	Functional independence	MRS	5	5	4	5	5	5
		BI	30	40	50	35	40	35
Two previous (b, d) and Environmental Factors (e)	Quality of live, anxiety, and depression	ECVI-38	74.95	49.30	51.04	57.18	45.94	51.64
		HADS	22	11	17	9	15	22

Abbreviations: BI: Barthel Index; BLS: Burke Lateropulsion Scale; ECVI-38: Quality of Life Scale for Stroke; FAC: Functional Ambulation Category; HADS: Hospital Anxiety and Depression Scale; ICF: International Classification of Functioning, Disability and Health; MRS: Modified Rankin Scale; PASS: Postural Assessment Scale for Stroke; POMA: Performed Oriented Movement Assessment; SCP: Scale of Contraversive Pushing; SF-MPQ: short form of the McGill Pain Questionnaire; TIS: Trunk Impairment Scale.

## Data Availability

Data are contained within the article.
